# Towards comparable quality-assured Azure Kinect body tracking results in a study setting—Influence of light

**DOI:** 10.1371/journal.pone.0308416

**Published:** 2024-08-09

**Authors:** Linda Büker, Michel Hackbarth, Vincent Quinten, Andreas Hein, Sandra Hellmers

**Affiliations:** 1 Assistance Systems and Medical Device Technology, Department for Health Services Research, School VI - School of Medicine and Health Sciences, Carl von Ossietzky Universität Oldenburg, Oldenburg, Germany; 2 Geriatric Medicine, Department for Health Services Research, School VI - School of Medicine and Health Sciences, Carl von Ossietzky Universität Oldenburg, Oldenburg, Germany; Imperial College London, UNITED KINGDOM OF GREAT BRITAIN AND NORTHERN IRELAND

## Abstract

Quality assurance in research helps to ensure reliability and comparable results within a study. This includes reliable measurement equipment and data-processing. The Azure Kinect DK is a popular sensor used in studies with human subjects that tracks numerous joint positions with the Azure Kinect Body Tracking SDK. Prior experiments in literature indicate that light might influence the results of the body tracking. As similar light conditions are not always given in study protocols, the impact needs to be analyzed to ensure comparable results. We ran two experiments, one with four different light conditions and one with repeated measures of similar light conditions, and compared the results by calculating the random error of depth measurement, the mean distance error of the detected joint positions, and the distance between left and right ankle. The results showed that recordings with similar light conditions produce comparable results, with a maximum difference in the median value of mean distance error of 0.06 mm, while different light conditions result in inconsistent outcomes with a difference in the median value of mean distance error of up to 0.35 mm. Therefore, light might have an influence on the Azure Kinect and its body tracking. Especially additional infrared light appears to have a negative impact on the results. Therefore, we recommend recording various videos in a study under similar light conditions whenever possible, and avoiding additional sources of infrared light.

## Introduction

Several hundred subjects are assessed repeatedly over a period of several years in large studies such as the “Sentinel fall presenting to the emergency department (SeFallED)” study [1]. Part of the SeFallED study is conducted in a laboratory environment, the so-called “gait lab”. During several functional assessments performed in the gait lab, the subjects walk on an instrumented treadmill (Motek Medical B.V., Amsterdam, the Netherlands) and are recorded by three Azure Kinect DK cameras (Microsoft, Redmond, USA). The resulting videos are processed with Microsoft’s Azure Kinect Body Tracking SDK to detect human poses, calculate joint positions, extract gait features, and determine gait stability. The Azure Kinect Body Tracking SDK uses the Azure Kinect DK’s 1-megapixel time-of-flight depth camera to extract the position and orientation of 32 key points/joints for each person standing in front of the camera. In general, body tracking can be performed either using a marker-based or a markerless capturing system. Markerless systems are less time-consuming than marker-based systems and can therefore be implemented more conveniently [2]. The Azure Kinect DK with its body tracking is a markerless system, that is widely accessible and relatively inexpensive. Multiple studies used and verified Azure Kinect’s body tracking in specific situations [3, 4], among other things also for gait analysis [5–7]. However, gait analysis with markerless systems comes with some challenges: in general, these systems may be biased by the databases they were trained on, whereby specific clinical movements (e.g. neurological disorders or amputations), specific populations (e.g. age or race), or specific setups (e.g. light conditions or camera angle) might influence the body tracking results [8]. Other challenges identified in literature using Azure Kinect’s body tracking is choosing the appropriate camera viewing angle [9], the influence of occlusions by, for example, walking aids on the accuracy of the body tracking when using a single camera [3], or the error in joint detection in comparison to gold standard systems [5]. These showed higher errors in feet or ankles, the relevant joints for gait analysis [5].

In general, when conducting and evaluating studies, it is important to maintain good scientific practice, for example, to ensure the reproducibility of the results of a measurement system [10, p. 17]. We analyzed the reproducibility of the Kinect’s body tracking before and found discrepancies between the results when using different processing modes available in the SDK or when using different computers for the processing [11]. At the same time, quality assurance in research incorporates reliable, and therefore comparable, measurement equipment, data-processing, and results. Therefore, this paper will focus on the analysis of the Azure Kinect’s body tracking in terms of comparable results, specifically on influential environmental factors, such as ambient light.

There is little literature that has analyzed the Azure Kinect DK itself or its body tracking regarding general accuracy or influential factors: Tölgyessy et al. investigated the accuracy of body tracking [12] as well as the depth sensor [13] in general. Yeung et al. [9] analyzed the influence of the camera angle on body tracking and Kurillo et al. [14] analyzed the spatial and random error of the depth sensor. Romeo et al. [15], on the other hand, studied four different factors influencing body tracking, namely distance to the camera, resolution of the depth camera, occlusions of the subject, and light. For the study of the influence of light, they used a 300 watt halogen lamp aimed directly at the subject which was either turned on or off, resulting in an illuminance of either 1750 lux or 10 lux one meter in front of the lamp. Their research showed that the detection accuracy of joint positions using Azure Kinect Body Tracking SDK decreases with increasing illumination. Their mean distance error was on average 1.44 times higher under full light than under low light conditions. Romeo et al. claimed that direct light exposure to the subject resulted in higher noise in the measured depth values. Tölgyessy et al. [13] also focused on the influence of light by studying the depth sensor’s accuracy itself in an outdoor environment. The wide field of view (WFOV) mode was extremely noisy, therefore unusable in outdoor environments. The narrow field of view (NFOV) showed better results. Nevertheless, there were many more invalid measurements outdoors than indoors, which means that this mode should also only be used outdoors to a limited extent.

In the setup of the aforementioned SeFallED study, it is not possible to ensure consistent light conditions at all times, especially since the repeated measurements take place spread over multiple years. Complete darkness is also not an option, as this would compromise the safety of the subjects while walking. However, since in accordance to Romeo et al. [15] and Tölgyessy et al. [13] different light conditions can influence quality assurance, these must be considered for the specific setup. In our opinion, however, the previous studies on the influence of light are not sufficient for our described use-case. The gait lab is indoors, which deems outdoors measurements irrelevant. Furthermore, Romeo et al. used a halogen lamp, which usually provides a considerable amount of infrared light.

Even though the camera should capture the infrared intensity invariant of the ambient light [16], we questioned whether the additional infrared light might have had an influence on their results. The time-of-flight camera illuminates the scene with near-infrared light and measures the delay between the emitted and the light reflected by the objects in the camera’s field of view [16, 17]. Saturated as well as low infrared signals may result in invalid depth measurements [17], thus it is unclear to what extent additional infrared sources in the room can affect Azure Kinect’s body tracking.

Therefore, the aim of this paper is to analyze the effects of different light conditions on the body tracking of the Azure Kinect by comparing the body tracking results of: 1. recordings under different light conditions and 2. multiple recordings under similar light conditions in the study setup.

## Materials and methods

The following sections describe the hardware and software used for the analysis in this work and the experimental setup applied for the recording of all videos. The analysis methods used are also described.

### Hardware and software

The recordings of all videos were taken with Microsoft’s Azure Kinect DK and the corresponding Azure Kinect Recorder, using the Azure Kinect SDK version 1.4.1. Thereby, the following parameters were set for every recording: Frame rate at 30 frames per second, depth mode at NFOV unbinned, RGB format at MJPG, RGB resolution at 2048 × 1536 pixels, RGB auto exposure on. The RGB camera firmware had version 1.6.110, while the depth camera firmware had version 1.6.80.

For the analysis, the *offline_processor* from the Azure Kinect Samples on GitHub [18] was used to save the body tracking results in a JSON file. The *offline_processor* used Microsoft Azure Kinect Body Tracking SDK version 1.1.2. All body tracking files were created with the processing mode Direct Machine Learning (DirectML) and on the same desktop computer. This computer ran Windows 10 and was equipped with an Intel Core i9–10980XE 18-Core processor running at 4.80 GHz and a NVIDIA GeForce RTX 3080 graphic card.

To keep a better overview, we focused on the analysis of 18 of the 32 joint positions provided by the Azure Kinect Body Tracking SDK. We selected the most relevant joints for gait analysis and further posture analyses. The analyzed joints are listed in Table 1.

**Table 1:** The joints from the Azure Kinect Body Tracking SDK included and excluded in our analyses. (For reference see [19]).

Included joints	Excluded joints
PELVIS	CLAVICLE_LEFT
SPINE_NAVEL	HAND_LEFT
SPINE_CHEST	HANDTIP_LEFT
NECK	THUMB_LEFT
SHOULDER_LEFT	CLAVICLE_RIGHT
ELBOW_LEFT	HAND_RIGHT
WRIST_LEFT	HANDTIP_RIGHT
SHOULDER_RIGHT	THUMB_RIGHT
ELBOW_RIGHT	HEAD
WRIST_RIGHT	NOSE
HIP_LEFT	EYE_LEFT
KNEE_LEFT	EAR_LEFT
ANKLE_LEFT	EYE_RIGHT
FOOT_LEFT	EAR_RIGHT
HIP_RIGHT	
KNEE_RIGHT	
ANKLE_RIGHT	
FOOT_RIGHT	

We used C++ with the Azure Kinect Sensor SDK and OpenCV for the analysis of the depth images and Python version 3.8.10 with numpy, math, pandas, json and matplotlib for further analysis of the Azure Kinect’s body tracking outcomes. The statistical analysis was conducted with SPSS V.29 (IBM, New York, USA).

During the recording of the videos, we measured the light conditions using an Adafruit TSL2591 (Adafruit Industries, New York, USA) light sensor. This sensor measures illuminance in lux as well as uniformly measured infrared intensity (unitless).

### Experimental setup

**Fig 1:**
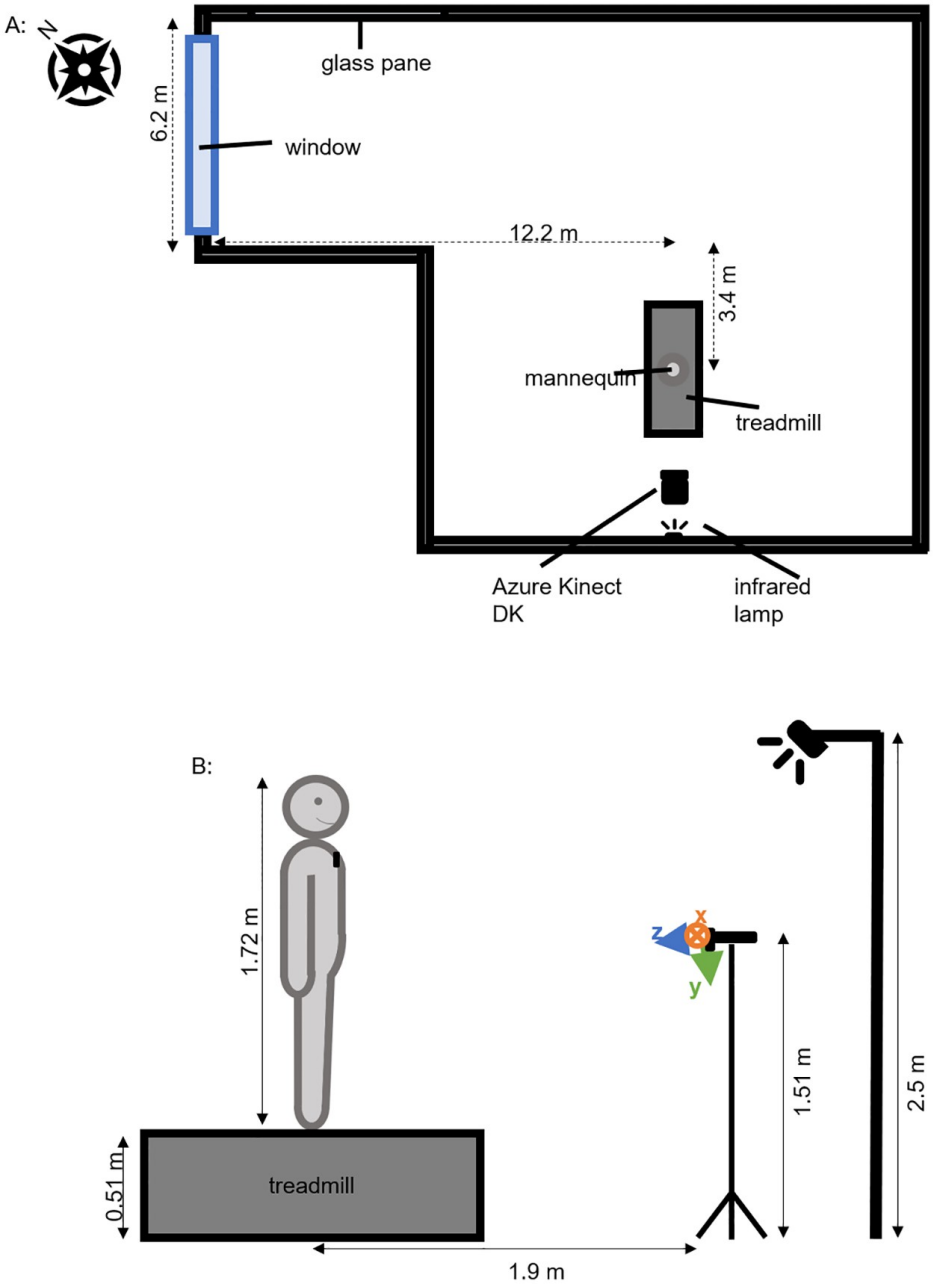
Sketch of the setup viewed from top and side. **A:** Setup from top view. Mannequin was standing on the treadmill facing the camera. An infrared light was installed in the background. The room’s window is not visible from the mannequin’s position. Next to the window is a glass pane to the room next door. At some positions of the sun, the sunlight is reflected in this pane when the blinds are up. Ceiling lights are not shown. **B:** Setup viewed from side. Mannequin was standing on a treadmill facing the camera with a light sensor installed on its left upper chest. In the background, an infrared light was installed. Not shown in the figure: the ceiling lights.

**Fig 2:**
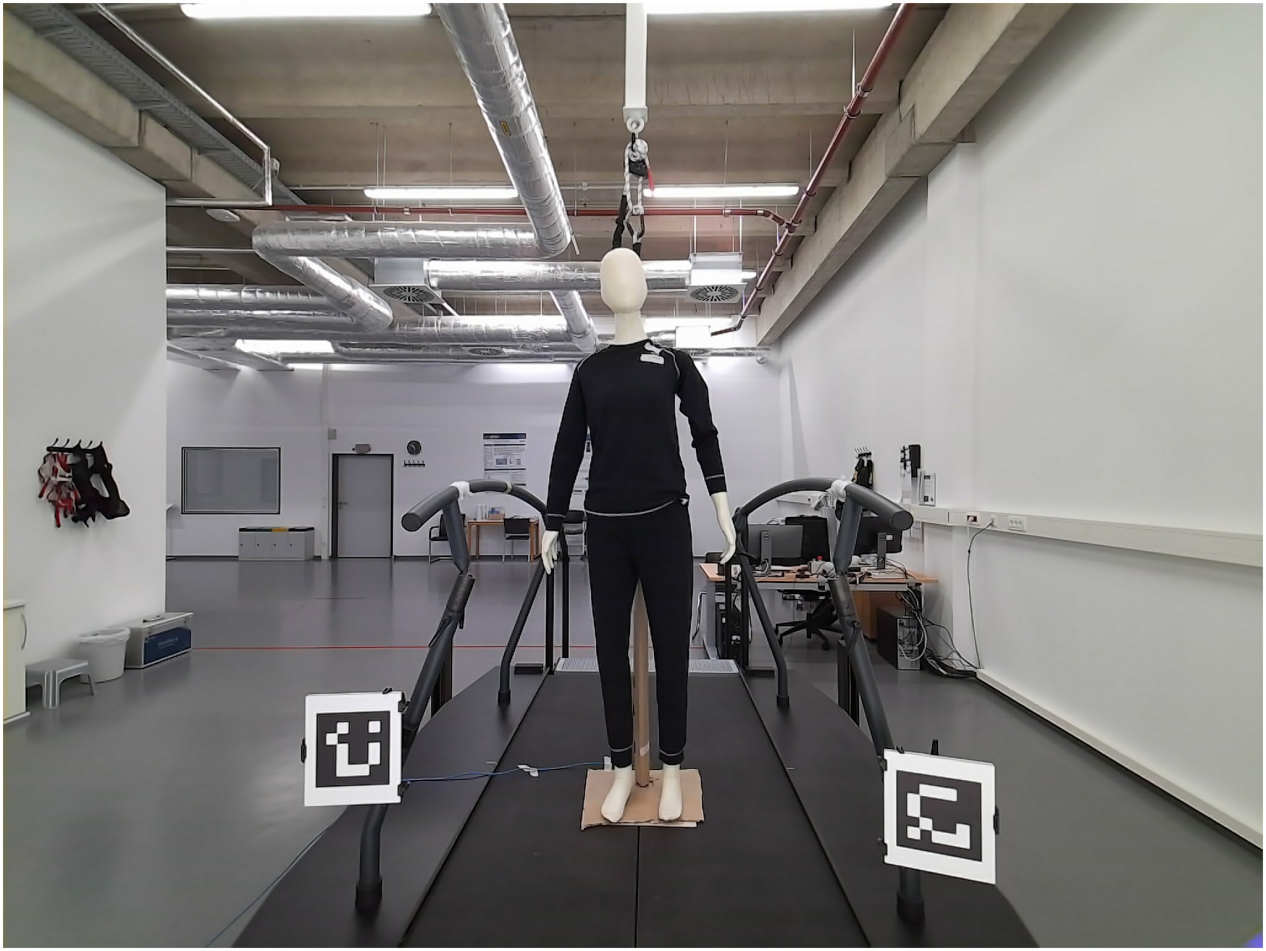
Mannequin standing on the treadmill in experiment 1 from the camera’s point of view in the SeFallED setup.

The experiments took place in the gait lab, the setup of the SeFallED study. A sketch of the floor plan of the gait lab together with the equipment used in our experiments is illustrated in Fig 1A. As described in the Section Introduction, one of the components of the setup is a treadmill of 0.51 m height. A mannequin of approximately 1.72 m height was placed on this treadmill to simulate a person and was fitted with black ski underwear made of cotton and nylon. The top and pants were made of the same material, this eliminates possible variations in the depth measurements because of different reflective properties of the materials. The mannequin was positioned such that all joints were visible within the camera’s field of view without any (self-)occlusions. For the SeFallED setup, three Azure Kinect DKs are positioned in front of the treadmill. However in our experiments, we used only the center camera facing the mannequin frontal. The height of this camera was approximately 1.51 m, positioned approximately 1.9 m in front of the mannequin. The camera’s view of the scene and the mannequin is shown in Fig 2. An additional infrared lamp was placed at a height of about 2.5 m behind the camera and aligned to illuminate the center of the mannequin (see Fig 1B). The lamp (ACC-07, Inkovideo, Hilden, Germany) has a range up to 80 m and a wavelength of 850 nm. This was done to integrate conditions with only infrared light and indoor lighting combined with additional infrared light. The standard lights consist of several fluorescent lights installed throughout the room as can be seen in Fig 2. The light sensor was placed in front of the mannequin’s left upper chest to measure the light intensity close to the mannequin. This location was chosen to have a central location frontal on the mannequin that was not in the direct vicinity of the analyzed body tracking joints, to minimize the chances of interference by the sensor. The gait lab has one large window facing the outside and can be darkened by blinds. However, this window is not visible from the actual setup because of the room’s shape (Fig 1).

For this study, we conducted two individual experiments: In experiment 1, we tested four different light conditions to simulate different ambient light and infrared intensities. In experiment 2, we compared five different recordings under similar light conditions.

Experiment 1 tested the most extreme light conditions possible in the gait lab, namely ceiling lights on or off, as well as an additional source of infrared light using the infrared lamp on or off to test a cross-section of the different ambient light conditions. For this experiment, the blinds were down to block out as much of the external natural light as possible.

Experiment 2, on the other hand, was designed to test the influences of light under typical conditions. A typical light condition during the measurements for the SeFallED study was chosen and five different measurements were made under this condition. For this experiment, the blinds were left up, since this is the typical condition in the room during measurements in the SeFallED study (during most of the year).

An overview of the different settings of the lights and blinds, as well as the measured visible and infrared light intensities, for the two experiments are listed in Table 2.

**Table 2:** Settings of the lights and blinds for all nine recordings, as well as the mean measured light (in lux) and infrared intensities (unitless).

Recording	Ceiling Light	Infrared Lamp	Blinds	Mean Illuminance (in Lux)	Mean Infrared Intensity
Experiment 1					
LightOff_IrOff	off	off	down	0	5
LightOff_IrOn	off	on	down	3	117
LightOn_IrOff	on	off	down	361	153
LightOn_IrOn	on	on	down	351	268
Experiment 2					
recording_1	on	off	up	293	118
recording_2	on	off	up	293	119
recording_3	on	off	up	292	119
recording_4	on	off	up	290	116
recording_5	on	off	up	290	114

As recommended by [13], the camera was warmed up for at least an hour before recording each video. Each video was recorded for 5 minutes, during which the mannequin and the camera were not moved.

The recorded depth data and the corresponding body tracking results are publicly available at our university’s data repository [20].

### Analysis

Prior to the analysis, we verified that the body tracking under the selected processing mode DirectML provided reproducible results on the used computer, by running the body tracking multiple times. Since all runs yielded the same results, all following analyses were performed on one body tracking run. As the body tracking showed a converging behavior in the first frames, we cut the first 60 frames of the body tracking results of every video in accordance with [11]. After cutting the first frames, no more converging behavior was observed in the remaining frames. This means, the frames from 61 till the last frame were analyzed. It should be noted, that the 5 minute videos were supposed to be 9000 frames long, however, the recorder actually recorded 9002 or 9003 frames.

To compare the noise of the camera’s depth measurement between the different videos, the random error *re* was calculated for each video using the pixels of three different areas: the mannequin’s belly, right knee, and right ankle with surrounding area, with sizes of 57x57 pixels, 15x15 pixels, and 25x25 pixels respectively. These sizes were determined visually by maximizing a square area without including other regions or the background (belly and right knee) and without including other regions, except some of the background (right ankle with surrounding area).

$$\begin{array}{c} re\left[p\right]=\sqrt{\frac{\sum_{t=61}^{f}{\left(d\left[p,t\right]-\bar{d[p]}\right)}^{2}}{\left(f-60\right)}}, \end{array}$$ (1)

where *t* is the frame in [61,…,*f*] with *f* being the last frame of the video *f* ∈ {9002, 9003} and $\bar{d[p]}$ the mean value over all measured depth values *d*[*p*, *t*] for all frames *t*, with *d*[*p*, *t*] measured at the pixel position *p*. Thereby, *p* is every pixel within the three areas mentioned before. Invalid depth values were not considered.

For all nine videos, the mean distance error (MDE) was calculated for every joint. According to Romeo et al. [15], MDE are the Euclidean distances of the coordinates of a joint to a corresponding centroid, averaged over all frames, and thus provides information about the intensity of the noise of the body tracking. To calculate the MDE, we first calculated the centroid *c* for every joint *j* in Table 1 and every frame *t* in [61,…,*f*] (with *f* ∈ {9002, 9003}) over a window of 2*N* + 1 frames:

$$\begin{array}{c} c\left[j,t\right]=(x_{c}\left[j,t\right],y_{c}\left[j,t\right],z_{c}\left[j,t\right]), \end{array}$$ (2)

with

$$\begin{array}{c} x_{c}\left[j,t\right]=\frac{1}{2N+1}\sum_{n=t-N}^{t+N}x\left[j,n\right], \end{array}$$ (3)

and analog for *y* and *z*. At frames *t* < *N* + 1 and *t* > *T* − *N*, the window length was shortened to the remaining frames. Similar to Romeo et al. [15], the window length parameter *N* was set to the frame rate, which meant *N* = 30 in our case. The Euclidean distance can now be computed as the squared error *se*:

$$\begin{array}{c} se\left[j,t\right]={\left(x\left[j,t\right]-x_{c}\left[j,t\right]\right)}^{2}+{\left(y\left[j,t\right]-y_{c}\left[j,t\right]\right)}^{2}+{\left(z\left[j,t\right]-z_{c}\left[j,t\right]\right)}^{2}, \end{array}$$ (4)

and averaged over time, which is then the mean distance error *MDE*:

$$\begin{array}{c} MDE\left[j\right]=\frac{1}{\left(f-60\right)}\sum_{t=61}^{f}\sqrt{se[j,t]}. \end{array}$$ (5)

Moreover, the distance *d* between the left and right ankle were calculated for each frame of each video:

$$\begin{array}{c} d_{t,ankles}\left(l_{t},r_{t}\right)=\sqrt{{\left(x\left[l,t\right]-x\left[r,t\right]\right)}^{2}+{\left(y\left[l,t\right]-y\left[r,t\right]\right)}^{2}+{\left(z\left[l,t\right]-z\left[r,t\right]\right)}^{2}}, \end{array}$$ (6)

where *t* is frame in [61,…,*f*] (with *f* ∈ {9002, 9003}), *l* is the left ankle and *r* is the right ankle. We calculated the distance between the ankles, often referred as stance width in static or step width in dynamic trials, as an example of relations between body parts relevant for posture analysis.

All datasets for random errors were tested for normal distribution with the Kolmogorov-Smirnov-Test. In case of non-normally distributed data, the random error of each pixel in the areas belly, right knee and right ankle were compared between light conditions (experiment 1) with an independent-samples Kruskal–Wallis test by ranks and between repeated recordings (experiment 2) with the related-samples Friedman’s two-way analysis of variance by ranks. One test was carried out for each area, comparing between all light conditions/recordings (see Tables 3 and 5, columns 1 and 2, for the list of conditions/repeats). In case of significant results, a pairwise comparison with Bonferroni correction was done. All tests were performed with a significance level of *p* ≤ 0.05.

**Table 3:** Minimum (Min), maximum (Max), median with interquartile range [IQR] of the random errors in mm for all light conditions in experiment 1 measured in the three areas belly, right knee, and right ankle with its surrounding area.

Area	Light Condition	Random Error per light condition (in mm)		
		Min	Max	Median [IQR]
Belly	LightOff_IrOff	1.11	1.61	1.20 [1.18; 1.25]
	LightOff_IrOn	1.27	2.00	1.37 [1.34; 1.43]
	LightOn_IrOff	1.11	1.60	1.20 [1.17; 1.25]
	LightOn_IROn	1.26	1.92	1.36 [1.33; 1.42]
Right Knee	LightOff_IrOff	1.36	1.82	1.44 [1.40; 1.48]
	LightOff_IrOn	1.52	1.93	1.60 [1.57; 1.64]
	LightOn_IrOff	1.35	1.87	1.41 [1.39; 1.46]
	LightOn_IROn	1.52	2.06	1.59 [1.56; 1.64]
Right Ankle + Surrounding Area	LightOff_IrOff	1.30	21.32	2.54 [1.61; 3.30]
	LightOff_IrOn	1.46	27.35	2.86 [1.83; 3.83]
	LightOn_IrOff	1.30	20.14	2.52 [1.62; 3.31]
	LightOn_IROn	1.46	20.07	2.91 [1.81; 3.81]

**Table 4:** Mean Distance Error values (in mm) for experiment 1 for all light conditions and all relevant joints, as well as median with interquartile range [IQR] of the MDE values of each recording. The lowest MDE per joint are in bold.

Joint	MDE per light condition (in mm)			
	LightOff_IrOff	LightOff_IrOn	LightOn_IrOff	LightOn_IrOn
ANKLE_LEFT	1.67	**1.53**	1.70	1.75
ANKLE_RIGHT	**1.76**	2.20	1.88	2.00
ELBOW_LEFT	1.34	1.65	**1.21**	1.54
ELBOW_RIGHT	**0.92**	1.15	1.02	1.24
FOOT_LEFT	**2.46**	3.01	2.80	3.04
FOOT_RIGHT	**3.31**	5.00	3.36	3.80
HIP_LEFT	**0.74**	0.84	0.83	0.89
HIP_RIGHT	**0.74**	0.91	0.86	0.92
KNEE_LEFT	0.90	0.98	**0.85**	0.98
KNEE_RIGHT	**0.83**	0.96	0.97	1.00
NECK	**1.11**	1.23	**1.11**	1.35
PELVIS	**0.62**	0.76	0.73	0.78
SHOULDER_LEFT	1.21	1.41	**1.09**	1.42
SHOULDER_RIGHT	**1.08**	1.22	1.16	1.40
SPINE_CHEST	**0.87**	0.94	**0.87**	1.04
SPINE_NAVEL	**0.68**	0.79	0.70	0.83
WRIST_LEFT	2.57	2.68	**2.34**	2.84
WRIST_RIGHT	**0.99**	1.95	1.69	2.00
Median [IQR]	**1.03 [0.84; 1.58]**	1.23 [0.95; 1.87]	1.10 [0.86; 1.70]	1.38 [0.99; 1.93]

**Table 5:** Minimum (Min), maximum (Max), median with interquartile range [IQR] of the random errors in mm for all recordings in experiment 2 measured at the three areas belly, right knee, and right ankle with their surrounding areas.

Area	Recording	Random Error per recording (in mm)		
		Min	Max	Median [IQR]
Belly	recording_1	1.18	8.00	1.29 [1.25; 1.34]
	recording_2	1.17	2.57	1.28 [1.25; 1.33]
	recording_3	1.19	2.55	1.29 [1.25; 1.33]
	recording_4	1.18	2.52	1.29 [1.26; 1.33]
	recording_5	1.19	2.48	1.29 [1.26; 1.33]
Right Knee	recording_1	1.46	1.89	1.57 [1.54; 1.63]
	recording_2	1.46	1.89	1.57 [1.53; 1.63]
	recording_3	1.46	1.89	1.56 [1.53; 1.62]
	recording_4	1.46	1.90	1.58 [1.53; 1.63]
	recording_5	1.47	1.88	1.57 [1.53; 1.63]
Right Ankle + Surrounding Area	recording_1	1.32	20.03	2.40 [1.57; 4.19]
	recording_2	0.00	24.95	2.41 [1.57; 4.23]
	recording_3	0.00	20.98	2.44 [1.57; 4.14]
	recording_4	0.00	24.50	2.43 [1.57; 4.17]
	recording_5	0.00	22.14	2.44 [1.58; 4.14]

For every analysis, the differences between the videos in each experiment are discussed. Furthermore, the results of the two experiments were evaluated together.

## Results

### Experiment 1—Different light conditions

The results for experiment 1 with four different light conditions are described in the following.

#### Random error

**Fig 3:**
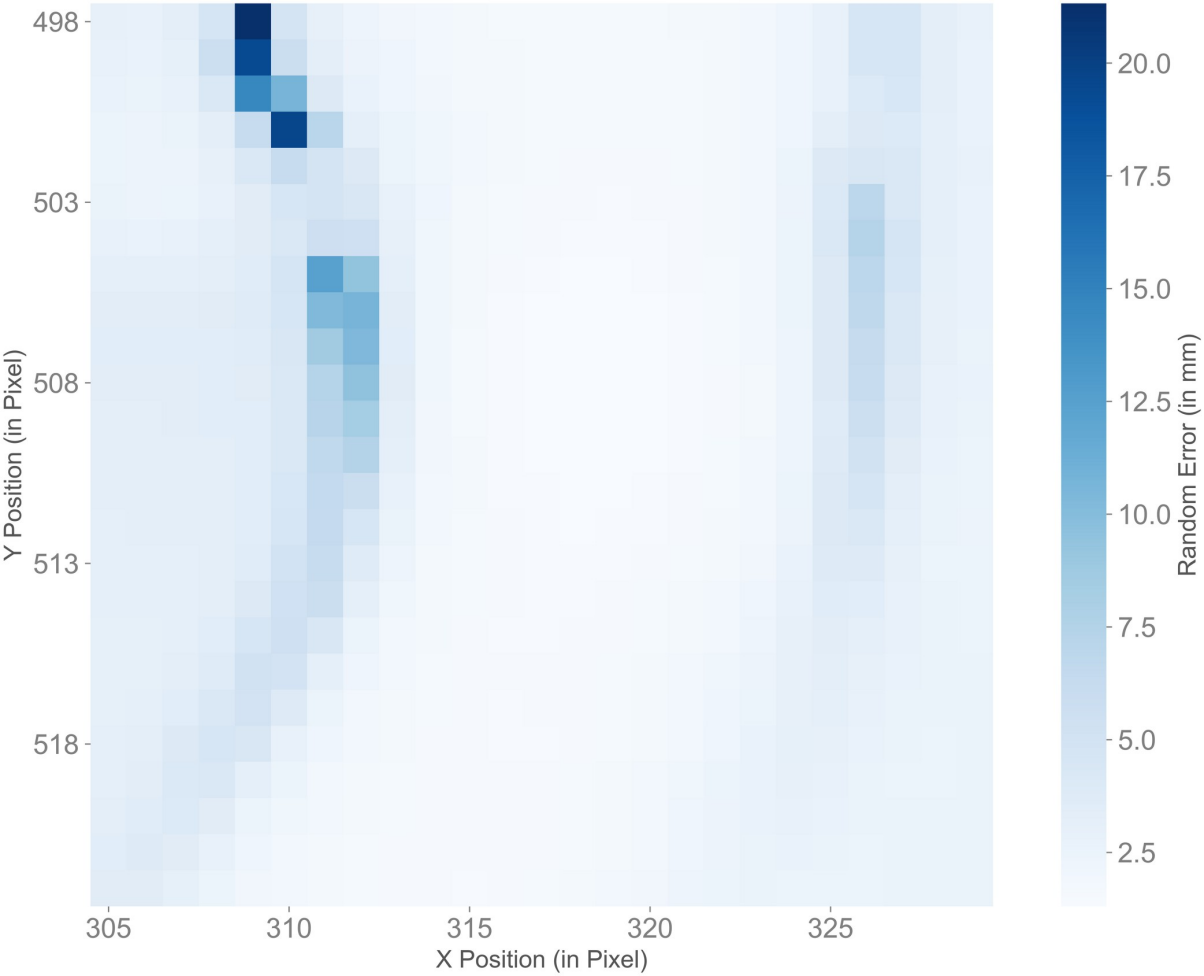
Sample visualization of random error values in experiment 1. Random error values for all pixels in the area of the right ankle with its surroundings at light condition LightOff_IrOff in experiment 1.

The random errors (see Eq (1)) were only calculated on valid depth values. The number of invalid depth values in comparison to the total number of pixels in the three different areas are shown in S1 Table. The belly and right knee had no or only few invalid values, the right ankle with the surrounding areas had around 50,000 invalid depth values for all light conditions, which is up to 1% of the total number of pixels. Table 3 shows the minimum, maximum, median and interquartile range of the random errors of all pixels in each of the three areas. The random error ranged from 1.11 mm (belly at LightOff_IrOff and LightOn_IrOff) to 27.35 mm (right ankle with surrounding area at LightOff_IrOn). It is of note, that the values for IrOn were always slightly higher than for IrOff. The random error values for the different light conditions showed significant differences between the four light conditions in all three areas (*p* < 0.001). The post-hoc pairwise comparison showed significant differences between the following light conditions: belly, between all light conditions (*p* < 0.001), except between LightOn_IrOff—LightOff_IrOff; both right knee and right ankle with surroundings, between all light conditions (*p* < 0.001), except between LightOn_IrOff—LightOff_IrOff and between LightOn_IrOn—LightOff_IrOn. Furthermore, the random errors in the belly area were lower than those in the right knee area, while these were lower than those in the area of the right ankle with the surrounding area. Fig 3 shows an example for the random errors in the area of the ankle with its surrounding for light condition LightOff_IrOff. This image shows an example of the distribution of random errors on the ankle, the surrounding area (treadmill) and the peripheral area.

#### Mean distance error

All MDE values calculated with Eq (5) for experiment 1 are listed in Table 4. The lowest values for the four different light conditions were between 0.62 mm and 0.78 mm (all for PELVIS, except light condition LightOn_IrOff, its lowest value was for SPINE_NAVEL). The highest values were between 3.31 mm and 3.80 mm, except for light condition LightOff_IrOn, here the highest value was 5.00 mm. The highest values consistently occurred on joint FOOT_RIGHT. The median values for videos with IrOff were 1.03 mm and 1.10 mm while the median values for videos with IrOn were 1.23 mm and 1.38 mm and therefore up to 0.35 mm higher.

#### Distance between left and right ankle

**Fig 4:**
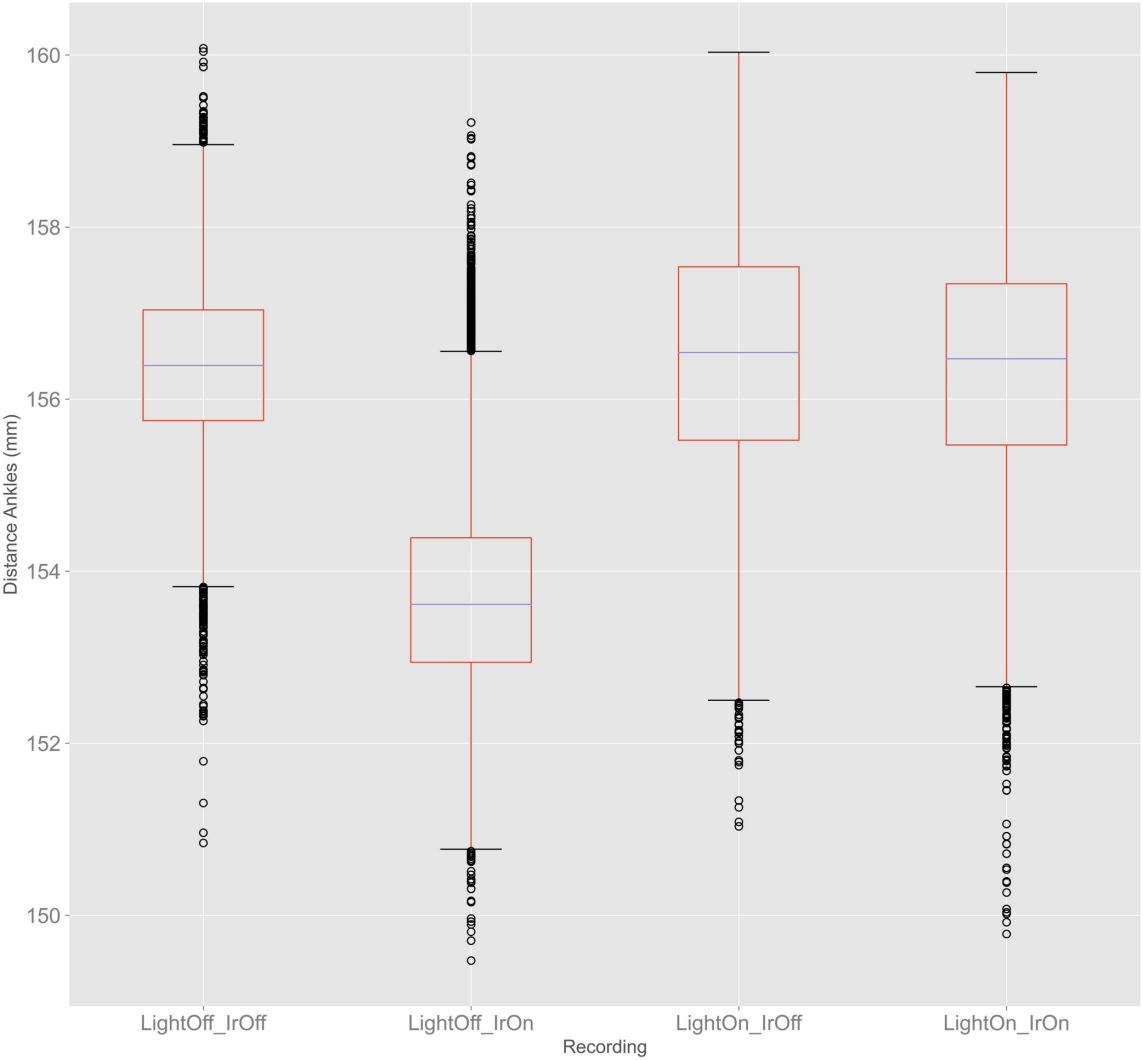
Boxplots for distances between left and right ankles for all light conditions in experiment 1.

Fig 4 shows the boxplots for the distances between left and right ankle compared for all four light conditions, calculated using Eq (6). The span between the maximum and minimum value, as well as the span between the third and first quartile were similar for all light conditions. LightOff_IrOn, however, had a median, first and third quartile approximately 3.0 mm smaller than the other light conditions.

#### Additional detected irregularities

**Fig 5:**
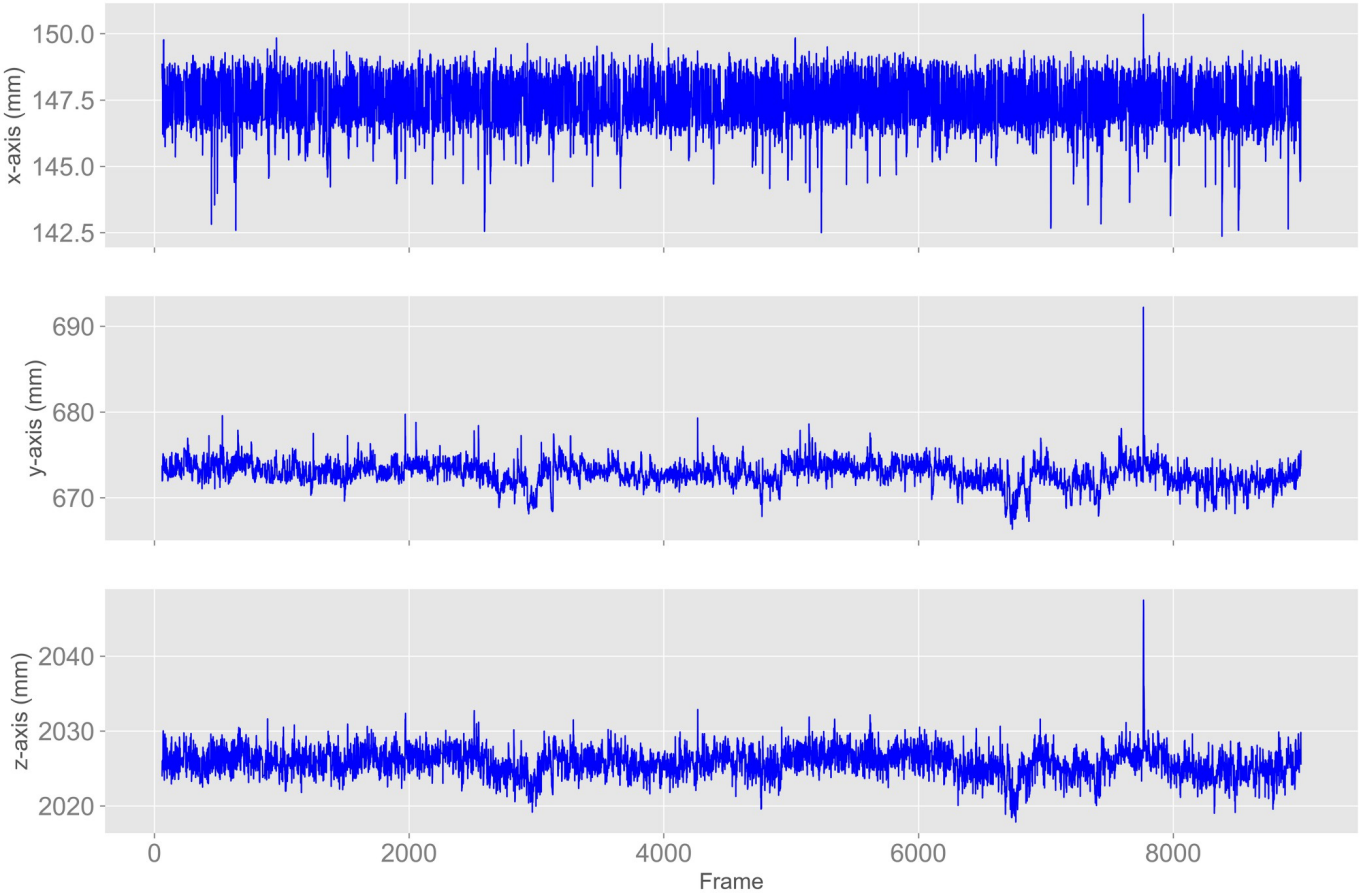
Peak in positions of light condition LightOn_IrOn for experiment 1. X-axis (points right of camera’s focal point), y-axis (points down), and z-axis (points forward) of the ANKLE_LEFT for experiment 1 and light condition LightOn_IrOn with a peak between frames 7000 and 8000.

**Fig 6:**
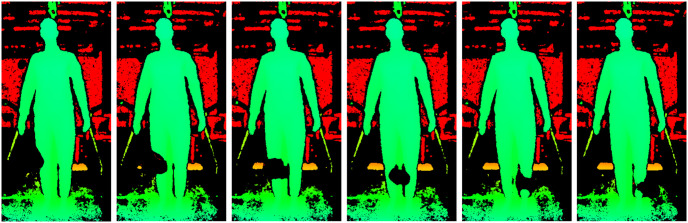
Black spot in light condition LightOn_IrOn for of experiment 1. Detail of the depth images of frames 7762 to 7767 of light condition LightOn_IrOn (experiment 1) with a black spot moving across the mannequin’s legs.

In the data of experiment 1, we noticed a peak in some joints that only occurred in LightOn_IrOn between frame 7000 and 8000. ANKLE_LEFT, for example, had a peak of almost 20 mm in the y-axis. Figs 5 and 6 show the plot for ANKLE_LEFT as well as the depth images of frames 7762 to 7767 with a black spot on the mannequin’s legs. This spot is black as the depth values were missing for this area. Over the time, this spot has moved across the depth image. No abnormalities could be identified in the corresponding color images.

Furthermore, when analyzing experiment 1, we noticed that WRIST_RIGHT of light condition LightOff_IrOff was detected more than 100 mm away from the other light conditions. An observation of the joint positions visualized in the point clouds of the different videos showed that the positions of the WRIST_RIGHT of the three light conditions LightOff_IrOn, LightOn_IrOff, and Light On_IrOn were recognized behind the actual wrist, while the position of the light condition LightOff_IrOff was recognized relatively well in the mannequin’s wrist.

### Experiment 2—Similar light conditions

The following sections describe the results for experiment 2, which consisted of five videos under similar light conditions.

#### Random error

**Fig 7:**
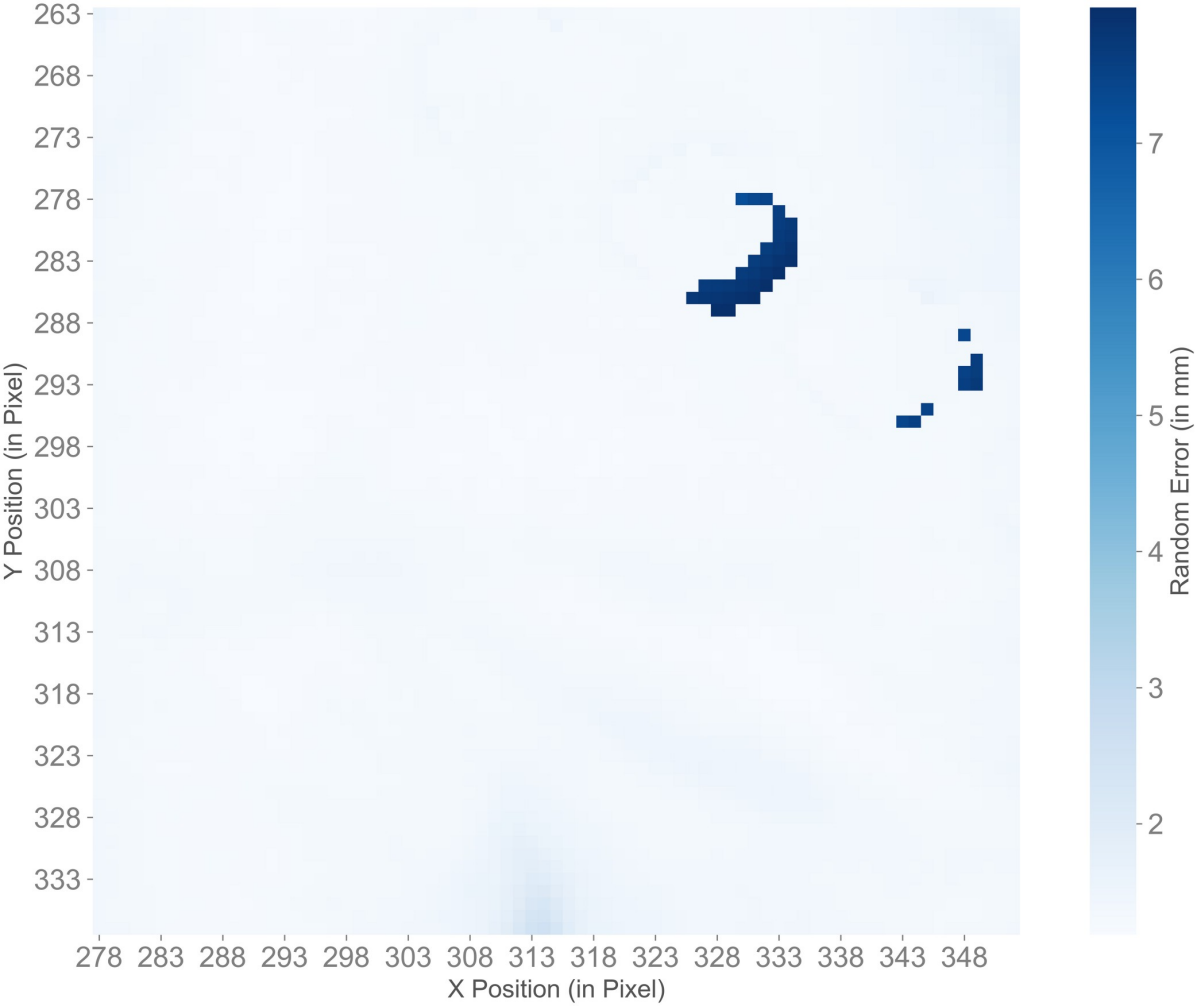
Sample visualization of random errors in experiment 2. Random errors for all pixels in the area of the belly in recording_1 of experiment 2.

In experiment 2, only valid depth values were included in the random error calculation (see Eq (1)). The number of invalid depth values ranged from approximately 0% for the belly and right knee to almost 9% for the right ankle with its surrounding area (see S2 Table). The minimum, maximum and median with interquartile range for the random errors for all pixels in each of the three areas are shown in Table 5. The random errors ranged from 0 mm (right ankle with surrounding area in recordings 2 to 5) to 24.95 mm (right ankle with surrounding area in recording_2). Upon further inspection of the minimum values of 0 mm, we found that valid depth values only existed in one frame for each corresponding pixels. This happened for eight pixels in total. While the right ankle with its surrounding area showed the highest random errors, the values for the belly area were the lowest. The random error significantly differed between the five recordings (belly & right knee: *p* < 0.001, right ankle with surroundings: *p* = 0.003). The post-hoc pairwise comparison showed differences between the following recordings: belly: all recordings (*p* < 0.001), except between recording_4—recording_5; right knee: between recording_1—recording_2, recording_2—recording_4, recording_2—recording_5, recording_3—recording_4 (all *p* < 0.001), between recording_1—recording_3 (*p* = 0.007) and between recording_4—recording_5 (*p* = 0.035); right ankle with surrounding: between recording_2—recording_3 (*p* = 0.003) and between recording_2—recording_4 (*p* = 0.031). Fig 7 shows an example for the random errors in the area of the belly for recording_2. This image shows an example of a cluster of higher random error values, while the other pixels in the area had lower random errors.

#### Mean distance error

All MDE values (see Eq (5)) for experiment 2 are listed in Table 6. PELVIS had the lowest MDE values for all recordings (between 0.84 mm and 0.90 mm). The highest values for all recordings were between 5.28 mm and 6.11 mm, all for joint FOOT_LEFT. The medians were between 1.41 mm and 1.47 mm and therefore had a difference of up to 0.06 mm. The first and third quartile had maximum differences of up to 0.11 mm and 0.21 mm, respectively.

**Table 6:** MDE values (in mm) for experiment 2 for all recordings and all relevant joints, as well as median with interquartile range [IQR] of the MDE values of each recording. The lowest MDE per joint are in bold.

Joint	MDE per recording (in mm)				
	recording_1	recording_2	recording_3	recording_4	recording_5
ANKLE_LEFT	**2.29**	2.43	2.42	2.30	2.37
ANKLE_RIGHT	2.10	2.10	2.07	2.16	**1.97**
ELBOW_LEFT	1.21	1.37	1.21	**1.18**	1.28
ELBOW_RIGHT	1.30	**1.26**	1.28	1.27	1.28
FOOT_LEFT	**5.28**	5.94	6.11	5.40	5.71
FOOT_RIGHT	4.59	5.05	5.03	4.79	**4.56**
HIP_LEFT	1.04	1.02	**0.98**	0.99	1.00
HIP_RIGHT	1.02	1.01	**0.98**	0.99	1.00
KNEE_LEFT	**1.47**	1.55	1.57	**1.47**	1.52
KNEE_RIGHT	1.63	1.69	1.66	1.63	**1.60**
NECK	2.29	2.30	2.22	**2.13**	2.24
PELVIS	0.90	0.87	**0.84**	0.86	0.87
SHOULDER_LEFT	**1.07**	1.22	1.22	1.10	1.20
SHOULDER_RIGHT	1.40	**1.33**	1.36	1.34	**1.33**
SPINE_CHEST	1.27	1.24	1.23	**1.15**	1.21
SPINE_NAVEL	1.04	1.01	0.99	**0.96**	0.99
WRIST_LEFT	2.49	2.67	**1.88**	2.02	2.26
WRIST_RIGHT	1.98	2.04	1.95	**1.85**	1.87
Median [IQR]	1.44 [**1.11**; 2.24]	1.46 [1.22; 2.25]	1.47 [1.21; **2.04**]	**1.41** [**1.11**; 2.10]	1.43 [1.20; 2.17]

#### Distance between left and right ankle

**Fig 8:**
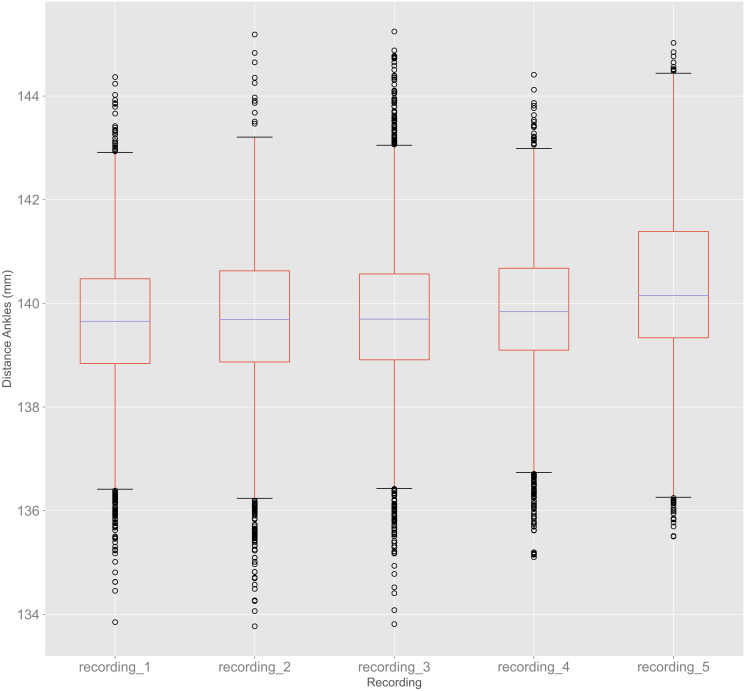
Boxplots for distances between left and right ankle for all recordings in experiment 2.

The boxplots for the distance between left and right ankle, calculated with Eq (6), compared for all five recordings are shown in Fig 8. One can see that there were only minor differences between the recordings. The highest median, for example, was only 0.5 mm higher than the lowest median. The span between third and first quartile was also similar for all recordings.

## Discussion

In this paper, we analyzed the effects of different light conditions on the body tracking of the Azure Kinect in the study setup of SeFallED by conducting two experiments. The differences and similarities between the videos are discussed below for Experiment 1—different light conditions and Experiment 2—similar light conditions. These results are then briefly related to each other in an Overall discussion, and finally the Limitations and recommendations for future research of the experiments are described.

### Experiment 1—Different light conditions

According to the manufacturer [21], the depth sensor’s random error is ≤ 17 mm. Kurillo et al. [14] found random errors between 0.6 mm and 3.7 mm at mean distances to the camera between 1 m to 5 m. Their measured random error at 2 m, which is similar to our distance, was 1.1 mm with the object being in the center of the camera’s view. The median of the random errors in experiment 1 were between 1.20 mm and 2.91 mm, which is 0.10 mm to 1.81 mm higher than the error found by Kurillo et al., but still considerably lower than the random error specified by the manufacturer. The maximum random errors at the right ankle with surrounding area, on the other hand, were higher than the value specified by the manufacturer. The high values occurred primarily in the peripheral area between the object (the mannequin) and the background (the treadmill) (see Fig 3). Kurillo et al. also indicated that higher random errors possibly occur at the object’s boundaries. Even though the random errors of all light conditions were low, it should be noted that errors were slightly higher with IrOn, which suggests that infrared light had a negative influence on the noise of the camera’s depth measurement. The fact that the light conditions with IrOff were always significantly different from the light conditions with IrOn, whereas the light conditions both with IrOff or both with IrOn were mostly not significantly different, supports the assumption that infrared light had an influence on the random error, while ambient light had no substantial influence.

With exception of the ANKLE_LEFT, the MDE values for IrOff were generally smaller than for IrOn with the same ceiling light (LightOn or LightOff). This means that the noise of the positions was slightly higher with additional infrared light than without. Although the differences in the MDE values were relatively small, this could indicate an influence of infrared light on Azure Kinect’s body tracking. Romeo et al. [15] found an average increase of MDE between light off and light on of a factor of 1.44, our results showed an increase of a factor of 1.22 between the lowest (LightOff_IrOff) and highest (LightOff_IrOn) MDE. These numbers are close to each other and the increase in our experiment resulted from switching on the infrared light. This supports our assumption that the infrared light in their halogen lamp may have had an impact on the body tracking.

The distances between the left and right ankle in experiment 1 showed no clear pattern indicating an influence of a specific light, as the spans between minimum and maximum, as well as three of the four medians were very similar to each other. Nevertheless, LightOff_IrOn had an average distance that was about 3 mm lower. According to the literature, normal step width variability ranges up to 25 mm, while higher values are considered excessive [22]. It is important to note that even though our error is a factor 10 smaller than the normal variability in step with, the detected difference in distance could be a meaningful change in elderly people who self-report difficulty in walking approximately one year after initially reporting no such difficulties [23]. Furthermore, the detected distance ranging approximately 9 mm must be considered, as this adds up to the 3 mm difference between light conditions. It can also be assumed that the accuracy of the body tracking will change in dynamic movements, which may cause accumulations of the errors for calculating step width. This means that a difference between the light conditions, even though way smaller than the variability in step width, cannot be neglected.

In total, it seemed that additional infrared light might have a negative influence on the depth images and body tracking. Light in general also might have an influence on analyses using the body tracking results.

### Experiment 2—Similar light conditions

In experiment 2, as in experiment 1, the medians of the random errors were higher than the error measured by Kurillo et al. [14], but still considerably lower than the error specified by the manufacturer [21]. Again, the maximum values at the right ankle with its surroundings exceeded the manufacturer’s specifications. The pairwise comparison of the recordings showed no clear pattern. However, it is noticeable, that 90% of the pairs were different in the belly area, while it was 60% of the pairs at the right knee and only 20% at the right ankle with surrounding areas. In contrast, the median of the random error was the smallest at the belly and the highest at the right ankle with surrounding area. Nevertheless, the maximum difference between absolute median values per area (0.0 mm to 0.04 mm) were statistically significant, but likely not clinically relevant. Especially considering that the mean marker distance error of marker-based motion capture systems like Vicon is usually greater than 0.04 mm [24].

The MDE values were very similar for each joint within the five videos of experiment 2. There is no visible structure in which recording the joint position had the most or least noise, suggesting that the different recordings had no effect on the noise of the joint position and thus on the MDE. Hence, the noise of the positions appeared to be relatively constant within different recordings under similar conditions. The highest MDE was a factor of 1.07 higher than the lowest MDE, which was a lower increase than found by Romeo et al. Nevertheless, this shows that not only the change of light condition can result in a different MDE, but also different recordings yield different MDE values.

With a maximum difference of 0.5 mm in the calculated median of the distances between left and right ankle, all five recordings showed similar results. This suggests that different recordings under similar light conditions lead to similar results in body tracking analyses.

In total, the results showed differences between different recordings under similar light conditions. However, these differences were small enough to suggest that they do not have a considerable impact on body tracking or on analyses of body tracking for most studies.

### Overall discussion

When comparing the results of the two experiments, one can see that in both experiments, the differences in random error between the videos were small, got bigger in MDE and were even bigger in the distance between left and right ankle. This means that in addition to the noise of the depth sensor, the Azure Kinect Body Tracking SDK adds further noise, which might add up in pose or movement analyses.

The results of experiment 2 showed that under similar light conditions the differences in multiple recordings were small enough to assume that different recordings have no substantial influence on the parameters observed in this paper. On the other hand, the results of experiment 1 showed that different light conditions lead to different results on the observed parameters. Especially additional infrared light appears to have a negative impact on the depth measurements and the body tracking. As the found differences were small and experiment 2 did not show exactly equal results, it is not clear whether these differences were actually caused by different light conditions or there was another influencing factor that was just not detected. Nevertheless, as a precaution, we recommend not to use additional infrared light when recording videos with the Azure Kinect. One should also try to record all videos of one study under similar ambient light conditions to enable best possible comparable results.

Looking at both experiments, we also observed that most of the considered invalid depth measurements occurred in the peripheral or the background area. This can be explained by the manufacturer’s information [17] that invalid depth pixels can occur due to too weak signals (background), or due to pixels containing a mixed signal from foreground and background (peripheral area). Amprimo et al. also raised awareness of interference problems at the border of the hand, which is why they had to adapt their method which detects hand key points with the help of MediaPipe on Azure Kinect DK videos [25]. However, in some of our videos several invalid depth measurements occurred on the belly or knee. The reason for this is unknown. In most cases were invalid depth measurements have occurred, the random error was also higher in the areas of invalid depth values, which means, there are areas with either no or worse depth measurements than other areas. Even though, invalid depth pixels and/or higher random errors can occur everywhere in a video, they appear to occur more often in peripheral and background areas. Therefore, one has to consider more imprecise depth measurements when analyzing these areas.

Moreover, we found additional detected irregularities in one of the nine videos: a peak in the position of some joints, probably due to a black spot moving across the depth image. The assumption is, that this black spot was a dust particle flying close to the camera lens. As the operating range for the used depth mode NFOV unbinned starts at 0.5 m [21], a dust particle too close to the depth camera might result in missing depth values at the respective spots in depth image. The body tracking could be influenced by the missing depth values resulting in a short change of position for the nearby joints. In future work, the occurrence of black spots can be further analyzed, however, in the case that dust is the issue, care should be taken to keep the study setup as dust-free as possible when conducting further studies to avoid peaks in the detection of joint positions.

It is also striking, that LightOff_IrOff was the only light condition under which the position of WRIST_RIGHT was recognized correctly. It is unclear why such a high error occurred in the other light conditions and why this behavior could not be observed with any other joint. Tölgyessy et al. analyzed the effects of different fabrics on Kinect’s depth signal noise and found that among other things the texture, transparency, porousness and reflectivity influence the noise [13]. This might also have played a role in the differences we found on the WRIST_RIGHT, as the top of the mannequin ends close to the wrist and different materials (black clothing and white fabric of the mannequin) meet. However, this cannot be proven with the available data. We performed a post-hoc analysis of the area around the WRIST_RIGHT to see whether the number of invalid depth values or the random error differed around this edge. We found almost no differences in the number of invalid depth values and only small differences in the random error (see S3 Table). Since we did not notice any abnormalities between the top and the hand, as well as between LightOff_IrOff and the other light conditions, we cannot say whether the clothing is the reason for the difference. As the Azure Kinect Body Tracking SDK is closed source, it is difficult to further examine this finding. Even though it is unclear, whether the different light conditions were responsible for this error, it is relevant to mention this finding, as a difference of up to 10 cm in different videos could lead to massive errors in further pose analyses.

### Limitations and recommendations for future research

Our experiments had the following limitations: By using a completely static mannequin instead of a real person, we were able to ensure that the different videos could be compared. However, it cannot be ruled out that the Azure Kinect Body Tracking SDK would have achieved different results with a real human. Furthermore, due to the nature of the mannequin and the needed comparability of the videos, we only analyzed a static scene and not dynamic movements as usually performed in the SeFallED study. It is unclear how body tracking behaves during movements. This should be investigated in another experiment. Thus, more static poses resembling a gait cycle could be tested first, which would possibly include poses with (self-)occlusion. The shape, texture and color of clothing may also have affected the accuracy of body tracking and could be analyzed in further studies. It should also be mentioned that the setup was slightly different in the two experiments, because the experiments were conducted on different days, this means that the positioning of the mannequin in relation to the camera and the pose of the mannequin itself varied to some extent.

To further analyze the influences of light, we recommend using multiple light sensors in future studies. Additional sensors on different body parts, especially around the joints close to the floor could reveal information about the relation between the local light conditions and the accuracy of the body tracking of different joints. Although, it should be noted the presence of a light sensor itself could also cause interference in the measurements. Furthermore, measuring the natural light entering the room is recommended to estimate its effect on the body tracking.

Since only one parameter configuration was tested, other frame rates and depth modes could be explored in further investigations. Finally, it should be mentioned that we did not determine a ground truth of the joint positions. This is due to the fact that a suitable method, marker-based infrared systems, would cause interference with the Azure Kinect infrared depth camera and thus the results would have been distorted [9]. Here, another suitable method should be sought to compare the positions found with the actual position, thus determining under which conditions the most accurate positions are detected.

## Conclusion

In conclusion, light may have a small influence on Azure Kinect DK and its body tracking. Our experiments show that additional infrared light increases the random error of the depth measurement of the Azure Kinect DK. Also the noise of the Azure Kinect Body Tracking SDK increases with additional infrared light. Furthermore, possible pose analyses can get different results on different light conditions. These effects appear to be small, but should be taken into consideration. Azure Kinect videos within a study should be recorded under similar ambient light conditions, without additional sources of infrared light, to generate the best possible comparable and consistent measurements.

## Supporting information

S1 Table Number of invalid depth values in the three areas belly, right knee, and right ankle with surrounding area for experiment 1. (PDF)

S2 Table Number of invalid depth values in the three areas belly, right knee, and right ankle with surrounding area for experiment 2. (PDF)

S3 Table Minimum (Min), maximum (Max), median with interquartile range [IQR] of the random errors in mm for all light conditions in experiment 1 measured at the top of the mannequin and the hand close to the WRIST_RIGHT. (PDF)
