# Supplementary material for: Towards comparable quality-assured Azure Kinect body tracking results in a study setting—Influence of light
Source: PLoS One. 2024 Aug 9;19(8):e0308416. doi: 10.1371/journal.pone.0308416 (PMC11315277; doi:10.1371/journal.pone.0308416)
Supplement: S1 Table — (PDF) [file pone.0308416.s001.pdf]

Area	Light Condition	Number of Invalid Depth Values (Total Number of Pixel)
Belly	LightOff_IrOff	0 (50,298,750)
	LightOff_IrOn	0 (50,304,375)
	LightOn_IrOff	142 (50,298,750)
	LightOn_IROn	0 (50,304,375)
Right Knee	LightOff_IrOff	0 (2,011,950)
	LightOff_IrOn	0 (2,012,175)
	LightOn_IrOff	0 (2,011,950)
	LightOn_IROn	517 (2,012,175)
Right Ankle + Surrounding Area	LightOff_IrOff	46510 (5,588,750)
	LightOff_IrOn	50,075 (5,589,375)
	LightOn_IrOff	42,535 (5,588,750)
	LightOn_IROn	50,094 (5,589,375)
