# Supplementary material for: Towards comparable quality-assured Azure Kinect body tracking results in a study setting—Influence of light
Source: PLoS One. 2024 Aug 9;19(8):e0308416. doi: 10.1371/journal.pone.0308416 (PMC11315277; doi:10.1371/journal.pone.0308416)
Supplement: S2 Table — (PDF) [file pone.0308416.s002.pdf]

Area	Recording	Number of Invalid Depth Values (Total Number of Pixel)
Belly	recording_1	685 (50,304,375)
	recording_2	0 (50,298,750)
	recording_3	7 (50,304,375)
	recording_4	0 (50,304,375)
	recording_5	2 (50,298,750)
Right Knee	recording_1	0 (2,012,175)
	recording_2	0 (2,011,950)
	recording_3	0 (2,012,175)
	recording_4	0 (2,012,175)
	recording_5	0 (2,011,950)
Right Ankle + Surrounding Area	recording_1	470329 (5,589,375)
	recording_2	469,539 (5,588,750)
	recording_3	476,861 (5,589,375)
	recording_4	482,652 (5,589,375)
	recording_5	481,393 (5,588,750)
