# Supplementary material for: Towards comparable quality-assured Azure Kinect body tracking results in a study setting—Influence of light
Source: PLoS One. 2024 Aug 9;19(8):e0308416. doi: 10.1371/journal.pone.0308416 (PMC11315277; doi:10.1371/journal.pone.0308416)
Supplement: S3 Table — (PDF) [file pone.0308416.s003.pdf]

Area	Light Condition	Random Error per light condition (in mm) Min	Max	Median [IQR]
top	LightOff_IrOff	1.28	1.62	1.33 [1.31; 1.43]
	LightOff_IrOn	1.52	1.80	1.56 [1.55; 1.64]
	LightOn_IrOff	1.28	1.67	1.34 [1.30; 1.45]
	LightOn_IROn	1.50	1.95	1.56 [1.51; 1.68]
hand	LightOff_IrOff	1.23	1.94	1.37 [1.29; 1.49]
	LightOff_IrOn	1.43	2.14	1.57 [1.49; 1.72]
	LightOn_IrOff	1.21	2.07	1.36 [1.29; 1.53]
	LightOn_IROn	1.44	2.41	1.61 [1.51; 1.80]
